# Role of lasers in restorative dentistry: a review and update

**DOI:** 10.3389/fdmed.2026.1941265

**Published:** 2026-09-21

**Authors:** Rashed A. Alsahafi, Heba A. Mitwalli, Basil Almutairi, Khalid S. Almulhim, Haitham AlQuorain, Turki Alshehri, Mohammed M. Gad, Abdulrahman A. Balhaddad

**Affiliations:** 1 Department of Restorative Dental Sciences, Umm Al-Qura University, College of Dentistry, Makkah, Saudi Arabia; 2 Department of Restorative Dental Sciences, College of Dentistry, King Saud University, Riyadh, Saudi Arabia; 3 Department of Restorative Dental Sciences, College of Dentistry, Imam Abdulrahman Bin Faisal University, Dammam, Saudi Arabia; 4 Department of Substitutive Dental Sciences, College of Dentistry, Imam Abdulrahman Bin Faisal University, Dammam, Saudi Arabia

**Keywords:** bleaching, bonding, caries, cavity, diagnosis, laser

## Abstract

**Objectives:**

This review aimed to summarize the most frequent clinical applications of lasers in restorative dentistry and provide the most recent updates.

**Methods:**

A comprehensive search of PubMed, Scopus, and WoS was conducted regarding the use of lasers in restorative dentistry. Many studies examined lasers' effectiveness in achieving clinically acceptable cavity preparation, removing caries, caries diagnosis, enhancing the bonding strength of direct and indirect restorations, tooth whitening, and treating dentin hypersensitivity.

**Results:**

Lasers were found to effectively prepare cavities and remove caries. The capability of lasers to achieve selective tissue removal makes them a conservative approach when removing caries. Lasers also reduce pain and anxiety for patients by minimizing vibration, noise, and the need for anesthesia. However, lasers’ drawbacks include imprecise cutting and slower speeds compared to conventional methods. In caries diagnosis, combining lasers with traditional methods has improved diagnostic accuracy. The effectiveness of lasers in enhancing the bonding strength of direct and indirect restorations remains a topic of debate. Additionally, while no significant advantages were found in using lasers for teeth whitening, lasers demonstrated greater efficacy than placebo or no treatment in the management of dentin hypersensitivity.

**Conclusion:**

Future research should focus on optimizing laser parameters in relation to restorative procedures, which can optimize the clinical benefits of using lasers.

## Introduction

In 1916, Einstein proposed the idea of stimulated emission. Einstein reported that the electrons could be excited to emit light with a particular wavelength (1). Lasers operate based on three fundamental components (Figure 1): the energy source, the active medium, and the optical resonator, each playing a crucial role in the generation and amplification of coherent light (2, 3). The energy source, often referred to as the “pump,” is responsible for exciting the atoms or molecules in the active medium. The active medium itself is the material that amplifies light through the process of stimulated emission, which can be a solid, liquid, or gas. The optical resonator, or cavity, enhances this light amplification process, typically consisting of two mirrors placed at either end of the active medium. One mirror is fully reflective, while the other is partially reflective, allowing photons to leap back and forth between the mirrors (2, 3).

**Figure 1:**
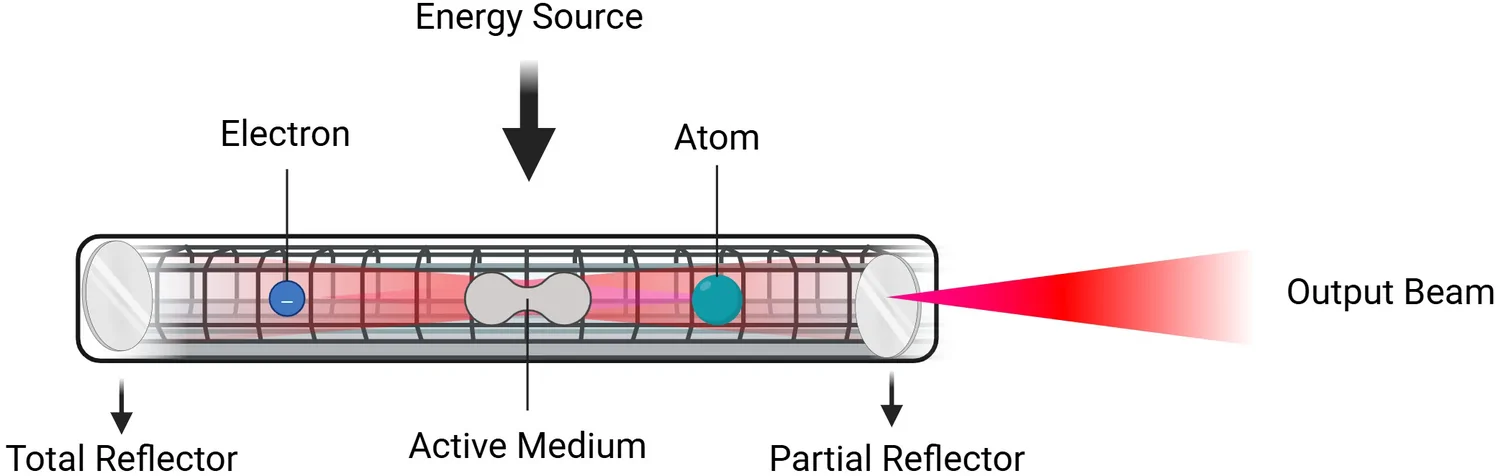
Schematic drawing shows the three fundamental parts of laser technology, which are the energy source, the active medium, and the optical resonator (total and partial reflectors). The figure was designed using Biorender.com.

This feedback mechanism enables the light to pass through the active medium multiple times, amplifying it further with each pass. The design of the optical resonator ensures that the emitted light is coherent, meaning the light waves are in phase and travel in the same direction. As the intensity of the light builds up, it eventually escapes through the partially reflective mirror, resulting in a focused laser beam. This intricate interplay of components allows lasers to produce powerful, narrow beams of light used in various applications, from medical procedures to telecommunications (4).

During the use of laser, certain characteristics are important to specify the effects on biological tissues, including wavelength, emission mode, peak power, and energy density. The wavelength needs to be determined carefully to specify the interaction with the chromophores of the tissues (5). Emission mode depends on the repetition rate and pulse duration, and according to these two factors, lasers could be operated in a pulsed or continuous wave. The pulsed wave is better than the continuous wave as it gives thermal relaxation time to the irradiated tissues (6). Different phenomena can occur due to laser-tissue interaction (Figure 2) (7). The interaction between lasers and biological tissues is a complex and well-investigated topic in biomedical optics, photomedicine, and laser surgery. The effects depend on laser parameters (wavelength, power, pulse duration, repetition rate) and tissue properties, such as absorption and scattering (8). The primary interactions can be classified as photothermal, photochemical, photomechanical, and photodisruption interactions (Figure 3).

**Figure 2:**
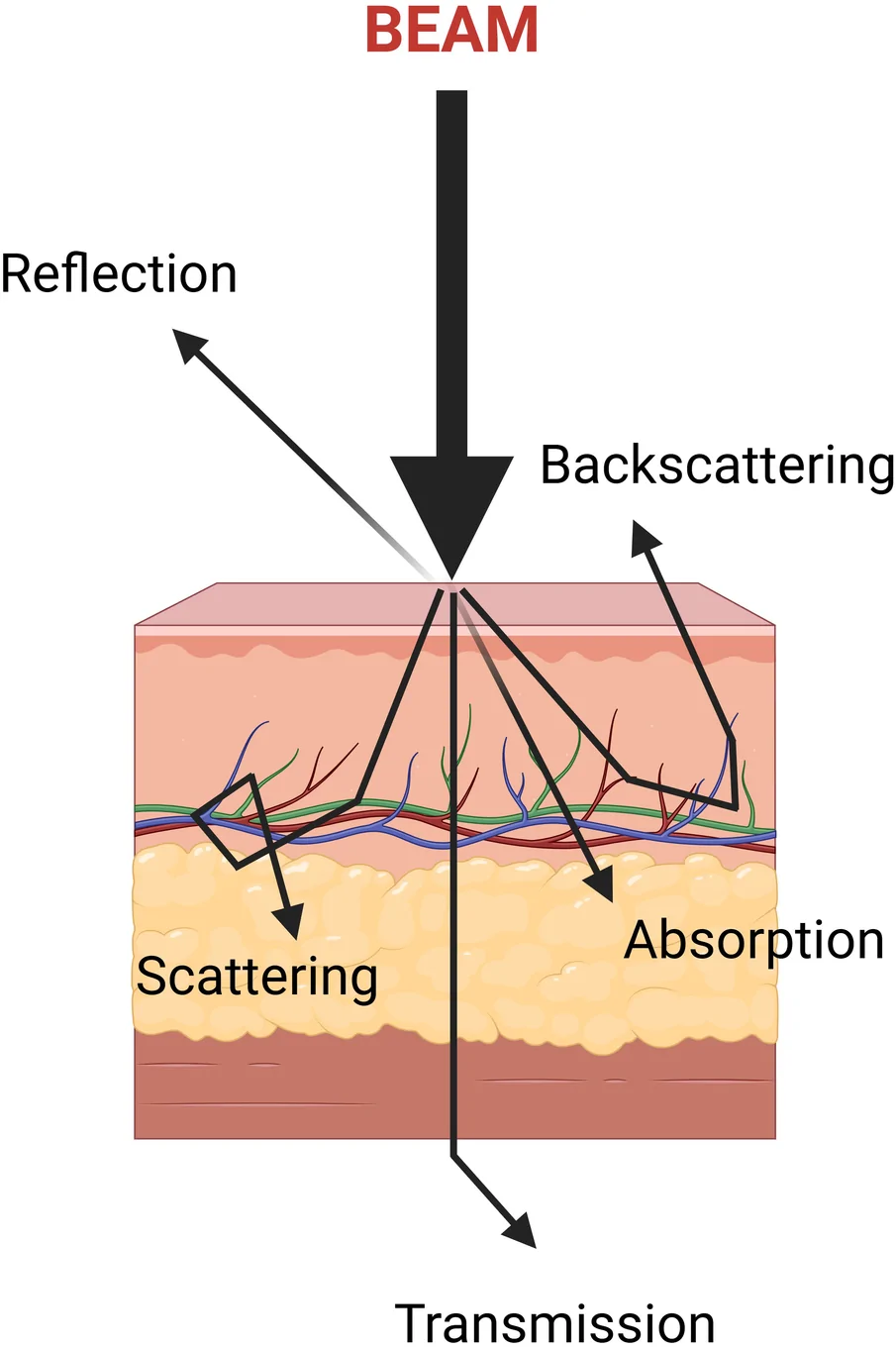
The tissue interaction with laser exposure can be experienced by laser transmission through the irradiated tissues, absorption, reflection, scattering, and backscattering. The figure was designed using Biorender.com.

**Figure 3:**
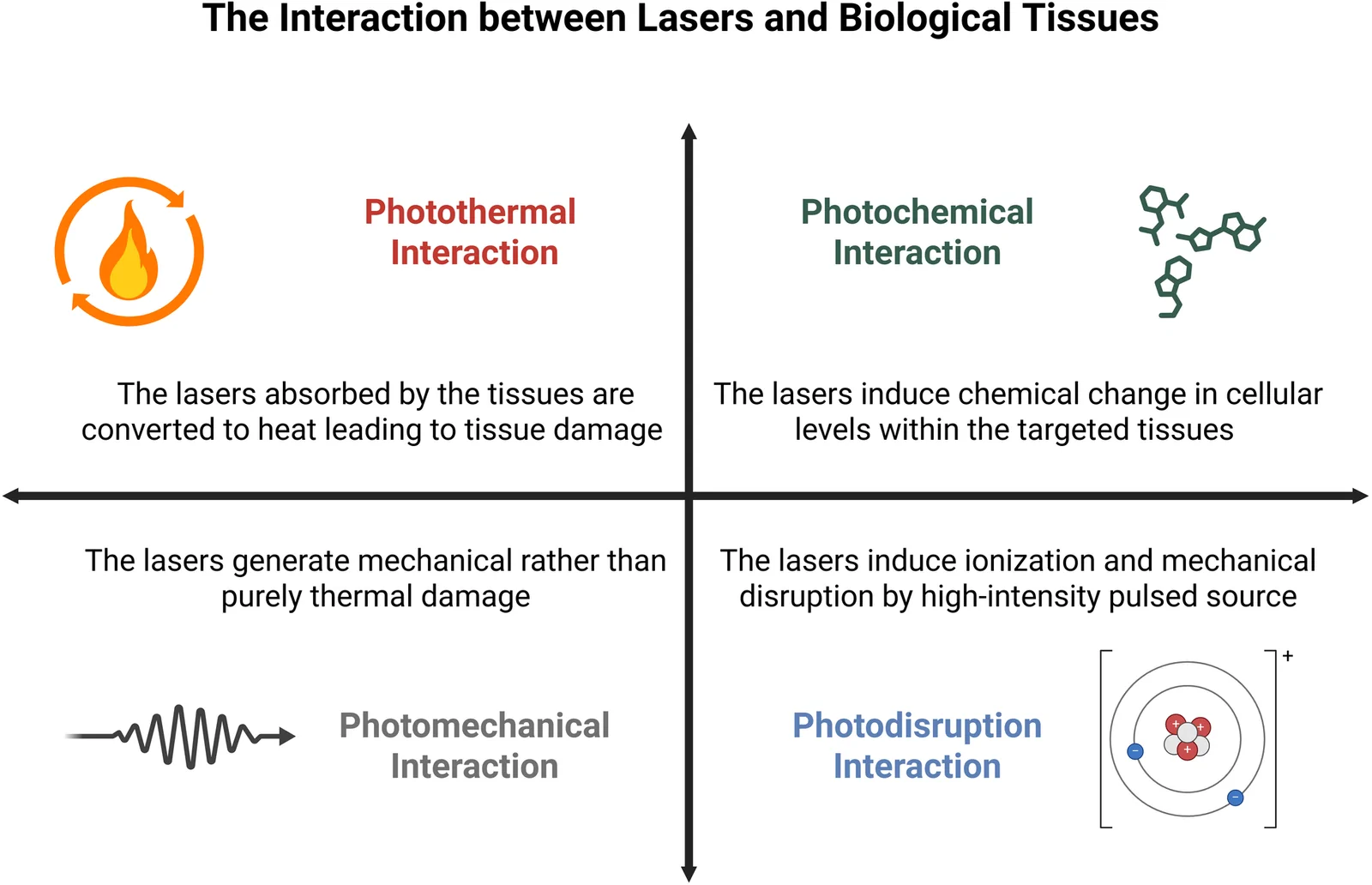
The mechanism of interaction between lasers and biological tissues. The figure was designed using Biorender.com.

Because of vibration and noise, attempts were made to find an alternative for rotary cutting instruments. A laser was used for the first time in restorative dentistry in 1964, when Goldman and others investigated the ability of a pulsed ruby laser to remove human caries (9). The main types of lasers used in restorative dentistry are erbium-doped yttrium aluminum garnet (Er:YAG), erbium, chromium: yttrium–scandium–gallium–garnet (Er,Cr:YSGG), neodymium-doped Yttrium Aluminum Garnet (Nd:YAG), argon, diode laser, and carbon dioxide (CO_2_) lasers (8, 10–13). This review provides a comprehensive overview of the use of lasers in restorative dentistry, exploring various aspects such as their applications, benefits, and limitations, as well as the latest advancements in technology and techniques. This review can be beneficial for clinicians and researchers alike, helping to minimize existing gaps in knowledge and practice.

## Search strategy

A narrative review approach was implemented to gather relevant studies on the use of lasers in restorative dentistry. The search was performed using databases such as PubMed, Scopus, and Web of Science, employing keywords including (laser OR “laser therapy” OR “diode laser” OR “Er:YAG”) AND (“restorative dentistry” OR “ restoration” OR “cavity preparation” OR “diagnosis” OR “caries removal” OR “microleakage” OR “bonding” OR “composite” OR “bleaching” OR “hypersensitivity” OR “erosion”) AND (application OR effectiveness OR outcome). The search was not restricted by a specific start date, and the final search was conducted in August 2026. Given the narrative nature of this review, study selection was not exhaustive but was guided by expert judgment to capture the breadth and clinical relevance of the available literature. Priority was given to more recent publications and to clinical trials, as these were considered most likely to reflect current clinical practice and provide clinically applicable evidence. Inclusion criteria focused on peer-reviewed articles in relation to laser applications in restorative dentistry. Exclusion criteria eliminated non-English publications and studies not directly related to restorative dentistry.

## Caries diagnosis

The use of laser technology for caries diagnosis has significantly advanced (14). This progress offers a non-invasive and measurable method for detecting dental caries. Contemporary innovations in laser-based technology, such as laser fluorescence (LF), optical coherence tomography (OCT), photothermal radiometry, and modulated luminescence (PTR-LUM), have been studied for their effectiveness in aiding caries detection.

Kanar and others in their *in vitro* study (15 extracted teeth per group) compared laser fluorescence readings with the International Caries Detection and Assessment System (ICDAS) scores and confirmed the results with histological tests. Results demonstrated that laser fluorescence demonstrates high accuracy in assessing caries removal across various techniques (15). However, another *in vivo* study by Perdiou and others compared the diagnostic reliability of laser-induced fluorescence (DIAGNOdent Pen) with light-induced fluorescence (VistaCam iX) and ICDAS visual criteria for occlusal caries on 139 molar teeth, and it was found that light-induced fluorescence and ICDAS showed more accurate diagnostic outcomes than laser-induced fluorescence devices for detecting occlusal caries (16).

Another promising technology in caries detection is OCT (17). An investigation by Shimada and others compared OCT caries diagnostic results across 111 sites of occlusal fissures of extracted teeth with a visual examination (18). The study concluded that OCT offers superior diagnostic accuracy in detecting subsurface carious lesions. Another study by Shimada and others evaluated the diagnostic accuracy of 3D OCT for detecting proximal caries in molars (19). They concluded that 3D OCT demonstrates higher sensitivity for diagnosing proximal caries in posterior teeth compared to intraoral digital radiography, especially for lesions extending to dentin. PTR-LUM is another promising laser-based technology (20). It uses laser-induced heat and light emissions to detect changes in the crystalline structure of tooth tissues. An *in vivo* clinical trial by Silvertown and others examined whether PTR-LUM values, as canary numbers, show a strong correlation between PTR-LUM values and ICDAS scores (20).

## Cavity preparation, caries removal, and cavity disinfection

The ablation of lasers against carious tissues is more effective and efficient, as the carious tissues have more water content that act as a good medium for lasers inside the tissues. Due to the differences in water content between sound and demineralized dentin, it is difficult to cut the carious dentin with the same threshold as the sound dentin (21). Accordingly, lower power is needed to cut the carious dentin, which is not efficient in producing precise cutting (22). However, the ability to use different powers in laser systems carries a huge advantage regarding the selectivity of tissue removal. Using a low power threshold allows the laser to cut the carious dentin without affecting sound dentin, which makes using the laser a conservative approach for cavity preparation (22).

The main concern of using lasers to prepare cavities was the potential to cause cracks. In 1998, Kataumi and others were the first to state that the Er:YAG laser could cause substructural cracks in dentin (23), which was supported by another study (24). Conversely, Müllejans and others conducted a study on ten extracted teeth. After the preparation, no signs of cracks were detected. The study does not provide strong evidence as it is an *in vitro* study (25). In 2002, a randomized clinical trial was conducted on 104 third molars with carious lesions scheduled for extraction and randomly divided into either pulsed Neodymium-doped Yttrium Aluminum Garnet (Nd:YAG) laser or conventional drill technique for treatment. After teeth extractions, they found that the pulsed Nd:YAG laser technique was not only effective in caries removal but also more conservative in removing normal enamel than the drilling technique (26).

These conflicting outcomes led investigators to investigate different lasers' parameters in cavity preparation. Therefore, an *in vitro* study investigated the effectiveness of Er:YAG laser with different powers to prepare extracted primary teeth (*n* = 8 per group). It has been found that the use of 10 Hz/200 mJ or 10 Hz/300 mJ power is effective and safe to perform the cavity preparation, while the use of 10 Hz/400 mJ power can lead to dentin cracks (27). A randomized clinical study that was conducted among twenty adult patients who had eighty carious lesions revealed that after four years of clinical service, there were no significant differences in the survival rate of resin composite restorations placed after cavity preparation using Er:YAG laser or burs (28). Another randomized clinical trial (*n* = 9 per group) revealed that laser irradiation following selective caries removal was associated with more dentin formation and more remineralization of affected dentin (29), likely because the laser sterilizes the prepared cavity, eliminating residual microbes and creating a cleaner environment for dentin formation. The disinfection of the tooth after cavity preparation using photodynamic therapy is another interesting topic in cariology. The activation of methylene blue using diode laser was found effective in reducing the load of cariogenic microbes in prepared cavities (30). Such findings may highlight the utilization of lasers in minimizing the risk of secondary caries by reducing the load of bacteria in the prepared cavity.

It is important for any cavity preparation approach to not induce any undesirable reaction to the dental pulp. No significant difference was reported regarding the inflammatory reaction and odontoblasts morphology between laser and the conventional technique (31). Besides, it was found that the laser-prepared tooth had a good healing capacity and was able to induce the formation of reparative dentin (32). It has been stated that the temperature induced by tooth preparation could be decreased by five degrees centigrade when the laser is used (11). Also, it was found that lasers decrease the amount of vibration one hundred times less than rotary cutting burs, causing less pain perception and pulpal reaction (33). To improve the selectivity of caries removal using lasers, a recent *in vitro* study examined the impact of different Er:YAG laser parameters on dentin preservation during caries removal (34). It was found that removing caries with an Er:YAG laser at 70 mJ energy, 300 µs pulse duration, and 20 Hz significantly preserved dentin compared with caries removal using burs or other laser parameters, suggesting that optimizing laser parameters is critical for improving clinical outcomes in caries removal (34).

Diode laser pretreatment has also been investigated as an adjunct to bioactive calcium silicate materials in vital pulp therapy. In a randomized controlled clinical trial evaluating indirect pulp capping in deep carious lesions, patients were allocated to receive either Biodentine alone or diode laser irradiation prior to Biodentine placement. Using cone-beam computed tomography to track dentin bridge formation over 12 months, both treatment groups demonstrated significant increases in dentin thickness; however, the group receiving diode laser pretreatment exhibited significantly greater dentin formation at both the 3- and 12-month follow-ups compared with Biodentine alone. Pulp vitality was maintained in all cases across both groups. Notably, patients in the laser-pretreated group reported significantly lower postoperative pain scores (35).

Patient perception has been measured in many studies. Patients always have the tendency to appreciate the treatment with less pain, stress, and time. Using rotary cutting burs is associated with vibration, pressure, heat, and noise that could be reduced or eliminated when lasers are used (36). Patients are less likely to get local anesthesia when laser is used (37). It has been reported that lasers are able to disrupt nerve terminals in the dentinal tubules between the odontoblast and myelin sheath, which could result in pain reduction (38).

## Marginal adaptation and microleakage

Restoration microleakage is associated with more plaque accumulation and bacterial infiltration that can cause hypersensitivity or recurrent caries. Marginal adaptation and microleakage after using the erbium laser were investigated in many studies. In 2011, an *in vitro* study among 21 extracted teeth found that the preparation by the Er:YAG laser had better marginal integrity than their bur counterparts (39). It has also been stated that when the etching follows a laser, it gives less microleakage (40). Yamada and others utilized the scan electron microscopy to investigate the microleakage and found the same efficiency for reducing microleakage between erbium-prepared cavities and etched-bur cavities (41). Other studies stated that when low laser power is used, there was no difference between the lased and bur-prepared cavities regarding the microleakage and the marginal integrity (42). Another study found that acid-etching alone produced significantly lower microleakage at the occlusal margin of Class V composite restorations than Er:YAG laser treatment or combined laser-plus-acid treatment. while no significant differences were observed among the tested groups at the gingival margin, indicating that laser irradiation is not a valid substitute for acid-etching on enamel (43). A recent systematic review and meta-analysis review of 11 studies revealed no substantial positive impact of using Er,Cr:YSGG on microleakage during cavity preparation, as additional acid etching was recommended following the laser treatment (44). A recent investigation on primary molars compared 2% chlorhexidine, diode laser, and Er:YAG laser as cavity disinfectants (*n* = 10 per group), finding that Er:YAG laser produced complete smear layer removal with wider dentinal tubules, while CHX left smear layer residues and the diode laser only partially removed it. Er:YAG also yielded significantly higher mineral content than the other two groups and resulted in the lowest microleakage, with the untreated control showing the highest. The study concluded that Er:YAG laser was the most effective disinfectant among the three, improving dentine morphology, mineral content, and restoration sealing (45).

## Lasers pretreatment of dentin preceding adhesion

Laser treatment via Er,Cr:YSGG has been demonstrated to improve the roughness of the dentinal surface and increase its surface area by accessing the patent dentinal tubules (46, 47). This enhancement allows adhesive monomers to diffuse more effectively into the tubules while simultaneously inhibiting the formation of smear layers. As a result, this treatment creates optimal dentin surfaces for strong bonding with resin composites and enhances the bonding techniques (46, 47). There are controversial results regarding the adhesion to laser-treated tooth structure. Using extracted teeth, Martínez-Insua and others reported that the adhesion to dental hard structure irradiated with Er:YAG laser is not as good as it can be done with conventional acid etching (*n* = 9 per group) (48). The same authors in another study found that acid etching was associated with higher shear bonding strength than Er:YAG laser treatment (49). Another study found no difference regarding the bond strength between lased-prepared teeth (*n* = 15 per group) without etching and lased-prepared teeth with etching when Er:YAG laser was used as a surface treatment (50).

Different results were reported in another investigation. Using extracted teeth, Gonclaves and others found that the micro-shear bond strength of Er:YAG-treated dentin was higher than the etch-treated surface regardless of the adhesive system (*n* = 10 per group). Using SEM, etch-treated samples showed an amorphous and uniform smear layer, while the Er:YAG-treated samples showed a lack of smear layer, irregular margins, and a huge number of exposed dentinal tubules (51). The same was reported regarding hybrid layer morphology in another *in vitro* study (*n* = 10 per group), where preparation with high-speed burs produced a thicker hybrid layer, 2.9 ± 1.71 μm, in comparison to Er:YAG-treated surface, 0.41 ± 1.00 μm to 2.06 ± 2.49 μm (52).

A recent systematic review and meta-analysis study concluded that there was no significant difference in the bonding strength between teeth treated with lasers and teeth with no treatment or with bur preparation (53). It has been stated that the bond strength following lasers' application revealed several challenges related to the reduced abilities of lasers to remove the peri-tubular dentin, difficulties in recognizing the presence of collagen matrix and the hybridization zone, and the presence of fewer resin tags. All these challenges were found to compromise the interaction between the collagen and the resin tags (53). However, it is important to state that lasers have the potential to enhance the adhesive strength of resin composites when applied to enamel that has recently undergone bleaching (54).

Lasers have also been used to improve the strength of repaired resin composite. It has been suggested that applying lasers on old resin composites can increase the micro-porous areas, which can improve the bonding between new and old resin composites (55). A recent investigation demonstrated that resin composite repair by treating old restoration with Er,Cr:YSGG laser is superior to roughening by the bur (56), another study demonstrated that air-abrasion (25.1 ± 6 MPa) was associated with higher micro-tensile bond strength than Er:YAG laser (21.2 ± 4.7 MPa) (57). The ability of Er,Cr:YSGG (58) and Er:YAG (59) in improving the bonding of resin composite to hybrid CAD/CAM ceramics was also reported. In a recent investigation that included 168 non-carious cervical lesions (NCCLs), NCCLs treated with Er,Cr:YSGG laser showed no significant difference in restoration retention compared to etched surfaces after one year of follow-up (60). In another investigation, a broader range of laser parameters was screened, examining diode lasers at several wavelengths to improve the micro-shear bond strength of dentine in extracted teeth. The 970 nm diode laser significantly increased bond strength compared to the control (no laser pretreatment), whereas the 445 nm and 660 nm diode lasers produced bond strength comparable to and lower than the control, respectively (61). The same study also included a randomized clinical trial in which non-carious cervical lesions (NCCLs) were treated with diode lasers at the three wavelengths above and compared to a control group. All groups showed comparable restoration retention, but the laser-treated groups showed significantly less staining around the restorations than the control (61).

## Lasers for cement bonding to ceramic materials and veneers De-bonding

All-ceramic restorations have become increasingly prevalent in contemporary dentistry, particularly lithium disilicate (LD) and zirconia-based ceramics. Laser irradiation has recently been investigated for its potential to enhance bond strength. Multiple laser systems have been evaluated, including Nd:YAG, Er:YAG, CO_2_, Er,Cr:YSGG, and Neodymium-doped yttrium orthovanadate (Nd:YVO₄) (62). In a study by Toyoda and others (63), the bonding performance of untreated zirconia, air-abraded zirconia, and zirconia treated (*n* = 12 per group) with ytterbium fiber laser was compared using two cements: composite resin and 4-Acryloyloxyethyl trimellitate anhydride/methyl methacrylate-tri-n-butylborane (4-META/MMA-TBB) resin. Post-thermocycling, laser treatment resulted in comparable bond strength to air-abrasion when 4-META/MMA-TBB was used, but significantly higher strength when bonded with composite resin.

Conversely, Uno and others (64) reported that CO₂ laser-treated zirconia demonstrated significantly lower bond strength (10.58 ± 1.64 MPa) compared to air-abraded zirconia (13.50 ± 1.34 MPa), but higher than untreated zirconia (5.80 ± 1.15 MPa). Fornaini and others (65) found no significant difference in bond strength between zirconia treated with fiber nanosecond-pulsed laser and untreated controls. In another investigation, Matei and others (66) observed that combining sandblasting with diode laser irradiation significantly improved bond strength (9.67 MPa) compared to sandblasting alone (7.71 MPa) or diode laser treatment (5.69 MPa) over a 12-month period (*n* = 10 per group). These heterogeneous findings suggest that laser treatment outcomes are highly variable and heavily dependent on the laser type and parameters.

For LD ceramics, Alavi and others (67) compared the shear bond strength following treatment with 9.6% hydrofluoric acid (HF), Nd:YAG laser, and CO_2_ laser (*n* = 12 per group). Their results confirmed that HF etching with silane yielded significantly higher bond strength (6.48 MPa) than either Nd:YAG (1.26 MPa) or CO_2_ laser (0.99 MPa), reinforcing HF as the standard protocol. More recently, Xu and others (68) evaluated femtosecond laser treatment and found that its bonding performance (6.7 MPa) was significantly lower than that of Monobond Etch & Prime (MEP), which achieved (8.9 MPa) after thermal cycling (*n* = 20 per group). In conclusion, the integration of laser surface treatment into routine clinical practice remains debatable. This hesitation primarily stems from the absence of standardized protocols and the significant variability in laser types and irradiation parameters (including beam angle) (69).

Laser technology has become an integral tool for many clinicians in the removal of ceramic veneers. Erbium lasers, such as Er:YAG and Er,Cr:YSG, are commonly utilized for this purpose due to their efficient interaction with dental materials (70). Clinical studies have validated the efficacy of laser-assisted debonding. An *in vitro* study evaluated the debonding strength of laminate veneers after Er:YAG laser application (71). The results indicated a significant decrease in bond strength, confirming the laser's effectiveness in veneer removal (*n* = 15 per group). These findings align with the systematic reviews, which concluded that erbium lasers have a substantial direct effect in reducing the shear bond strength of ceramic veneers, allowing for safe removal without harming the underlying tooth surface (72). The most common laser parameters for veneer debonding included energy levels ranging from 0.15 to 0.36 J, power settings between 1.5 and 5.4 Watts, and frequencies ranging from 10 to 30 Hz, with Er:YAG being the most used laser (73). A recent study investigated the impact of different laser parameters on veneer debonding and the resulting temperature rise at the tooth-veneer interface (74). Easier debonding was achieved with higher laser power (up to 3.0 W), minimal defocus (0 mm), and a 1-picosecond pulse duration. However, more favorable outcomes in terms of clean fracture surfaces and minimal adhesive residue were observed with a combination of 2 W power, 1 ps pulse duration, and 0.6 mm defocus. With regard to temperature rise, lower power combined with increased defocus (0.6–1.0 mm) and a 1-picosecond pulse duration kept the temperature increase safely below the 42.5°C threshold (74).

## Lasers in bleaching, dentin hypersensitivity treatment, and prevention of erosion progression

There are other uses of laser in restorative dentistry, including bleaching, dentin hypersensitivity treatment, and prevention of erosion progression. Three laser types are commonly used and FDA-approved for these applications: the argon laser (488/514 nm), the CO_2_ laser (9,300–10,600 nm), and the 980 nm gallium aluminum arsenide (GaAlAs) diode laser (75). A systematic review and meta-analysis study revealed that laser, such as diode and argon lasers, has no additional benefits in sensitivity reduction or improving color whitening compared to bleaching with no laser (76). Sgolastra and others conducted a systematic review to investigate the efficiency of lasers in treating dentin hypersensitivity. After analyzing thirteen studies, they concluded that Er:YAG, Nd:YAG and GaAlAs are all effective in reducing dentin hypersensitivity (77). The same observations were obtained in another systematic review (78), with most of the studies using a diode laser in continuous mode with an energy density ranging between 2.5–128 J/cm^2^, power settings between 0.15 and 3 Watts, and an exposure time between 10 and 120 s. However, in another systematic review, there was no significant difference between lasers in reducing dentin hypersensitivity compared to any other in-office desensitizing agents (79), suggesting that lasers are effective in reducing tooth sensitivity, but with no superiority over conventional approaches.

Moreover, the results of an umbrella review of nine systematic reviews indicated that the use of lasers effectively diminished dentin hypersensitivity when compared to either a placebo or no treatment, both in the short term and over an extended period. Additionally, there were no notable variations in effectiveness among the different types of lasers used (80). A recent study highlighted the benefits of combining lasers with other desensitizing agents, concluding that combining a 980 nm diode laser with amine fluoride was more effective for pain control than amine fluoride alone (81). Similarly, combining bioactive glass with Nd:YAG laser treatment was found to be significantly more effective in reducing sensitivity after scaling and root planing compared with each treatment alone (82). For the prevention of erosion progression, it has been demonstrated that the combination of laser and fluoride application could be an effective approach to stop the progression of enamel erosion (83, 84). However, using laser to stop erosion progression in dentin is not recommended, as it might result in more surface loss (83).

## Future perspectives and conclusion

There is no absolute indication or contraindication to using lasers in restorative dentistry. But in general, lasers could be indicated in any restorative procedure that has been discussed in this paper. Few drawbacks were reported that could limit the use of lasers. For example, lasers are associated with pulpal floor irregularities during cavity preparation. This limitation could eliminate the use of amalgam as a restoration. Overcoming this problem can be achieved by using resin composite as a final restoration or finalizing the cavity with hand instruments or rotary cutting burs. Cost and difficulty of use could deter the dentists' ability to consider lasers even though they are convinced that lasers are better. While one of the disadvantages of lasers is low speed, lasers have less noise and less need for anesthesia, which can make the patient more comfortable. In addition, Lasers are associated with less heat and vibration that keep the pulp more stable. While it is still difficult for lasers to replace the conventional approach of cavity preparation, the use of laser in selective caries removal could provide a huge advantage in removing infected dentin in cavities with deep carious lesions without affecting the sound tooth structure. Future investigations may focus on optimizing the use of lasers in such clinical situations to allow for a more conservative approach during caries removal and cavity preparation.

While there is controversy surrounding the use of lasers to minimize microleakage and enhance resin composite bonding, it is important to highlight their promising applications in caries diagnosis and the bonding of ceramic materials. In caries diagnosis, laser technologies, such as OCT, appear to improve the accuracy of dental caries detection. These findings underscore the need for further advancements to address the drawbacks related to cost and certain technical challenges. Additionally, the use of lasers for surface treatment of ceramic restorations warrants further investigation. The mixed results reported in the literature indicate a need for optimization and standardization regarding the types of lasers and ceramic materials used. In conclusion, this review summarizes the applications of lasers in restorative dentistry, with main advantages related to their minimally invasive interference and selectivity in tissue removal. Given their potential to revolutionize various aspects of restorative dentistry, further optimization and standardization are essential to maximize clinical benefits.

A critical challenge across the reviewed literature is the lack of standardized laser parameters, including wavelength, power output, pulse duration, energy density, and application technique. Studies investigating similar clinical applications often used obviously different settings, making direct comparisons of outcomes difficult and contributing to the conflicting results reported for procedures such as caries removal, bonding, and ceramic surface treatment. This variability not only obscures the analysis of existing evidence but also limits the ability of clinicians to translate research findings into consistent, reproducible clinical protocols. Without standardized parameters tailored to specific laser types, tissue targets, and clinical indications, the risk of limited or sub-optimal outcomes remains a concern. Establishing evidence-based parameter guidelines, potentially through consensus protocols or well-designed clinical trials, would be an important step toward improving the reliability and reproducibility of laser applications in restorative dentistry, and would eventually support their broader and safer clinical use.

It is also essential to emphasize the evidence level supporting the applications of laser discussed in this review. Most of the studies addressing microleakage, bonding, and ceramic surface treatment are *in vitro* investigations, which while important in proving potential benefits under controlled conditions, do not completely reflect the complexity of the oral environment. On the other hand, clinical evidence via randomized clinical trials addressing caries removal, dentin hypersensitivity, and pulp diagnostics deliver stronger applicability to actual clinical practice. The imbalance between *in vitro* evidence and clinical applicability should be considered when interpreting the conclusions of this review, as results observed at the laboratory level do not necessitate clinical validation. This observation highlights the importance of having future research to prioritize well-designed clinical trials, particularly for applications currently supported mainly by *in vitro* data to strengthen the overall quality of evidence in this field.

Finally, it is important to highlight that this review, as a narrative review, has some limitations. One of the limitations is subjectivity, as the selection of studies and interpretation of findings often reflect the author's perspectives rather than an objective analysis. Additionally, the quality of the included studies may vary significantly, complicating the ability to draw robust conclusions. Another drawback is the absence of quantitative analysis, which limits the assessment of evidence strength. Lastly, narrative reviews may focus more on recent studies, potentially overlooking important foundational research.
